# Improved image reconstruction of ^89^Zr-immunoPET studies using a Bayesian penalized likelihood reconstruction algorithm

**DOI:** 10.1186/s40658-021-00352-z

**Published:** 2021-01-19

**Authors:** Julian Kirchner, Joseph A. O’Donoghue, Anton S. Becker, Gary A. Ulaner

**Affiliations:** 1 Department of Radiology, Memorial Sloan Kettering Cancer Center, New York, NY USA; 2 Department of Diagnostic and Interventional Radiology, University Dusseldorf, Medical Faculty, Dusseldorf, Germany; 3 Department of Medical Physics, Memorial Sloan Kettering Cancer Center, New York, NY USA; 4 Department of Radiology, Weill Cornell Medical College, New York, NY USA; 5 Molecular Imaging and Therapy, Hoag Family Cancer Institute, Newport Beach, CA USA

**Keywords:** ^89^Zr-immunoPET, Reconstruction algorithms, Q.Clear, BSREM, OSEM

## Abstract

**Purpose:**

The aim of this study was to evaluate the use of a Bayesian penalized likelihood reconstruction algorithm (Q.Clear) for ^89^Zr-immunoPET image reconstruction and its potential to improve image quality and reduce the administered activity of ^89^Zr-immunoPET tracers.

**Methods:**

Eight ^89^Zr-immunoPET whole-body PET/CT scans from three ^89^Zr-immunoPET clinical trials were selected for analysis. On average, patients were imaged 6.3 days (range 5.0–8.0 days) after administration of 69 MBq (range 65–76 MBq) of [^89^Zr]Zr-DFO-daratumumab, [^89^Zr]Zr-DFO-pertuzumab, or [^89^Zr]Zr-DFO-trastuzumab. List-mode PET data was retrospectively reconstructed using Q.Clear with incremental *β-*values from 150 to 7200, as well as standard ordered-subset expectation maximization (OSEM) reconstruction (2-iterations, 16-subsets, a 6.4-mm Gaussian transaxial filter, “heavy” *z*-axis filtering and all manufacturers’ corrections active). Reduced activities were simulated by discarding 50% and 75% of original counts in each list mode stream. All reconstructed PET images were scored for image quality and lesion detectability using a 5-point scale. SUV_max_ for normal liver and sites of disease and liver signal-to-noise ratio were measured.

**Results:**

Q.Clear reconstructions with *β =* 3600 provided the highest scores for image quality. Images reconstructed with *β-*values of 3600 or 5200 using only 50% or 25% of the original counts provided comparable or better image quality scores than standard OSEM reconstruction images using 100% of counts.

**Conclusion:**

The Bayesian penalized likelihood reconstruction algorithm Q.Clear improved the quality of ^89^Zr-immunoPET images. This could be used in future studies to improve image quality and/or decrease the administered activity of ^89^Zr-immunoPET tracers.

**Supplementary Information:**

The online version contains supplementary material available at 10.1186/s40658-021-00352-z.

## Introduction

There are an increasing number of investigations of immunoPET for multiple clinical applications, including targeted imaging of HER2, CD38, PD-L1, CA9, PSMA, and others [1–7]. Zirconium-89 (^89^Zr) is most often chosen as the imaging radionuclide for immunoPET due to its favorable physical and chemical properties, including a 78-h half-life that is compatible with the relatively long times (on the order of a week) required to achieve optimal target-to-background differentials with antibody tracers [1, 4, 8]. However, ^89^Zr is not ideal, as there are issues with both patient radiation dose and image quality.

The positron yield of ^89^Zr is only 23% and it emits a (non-coincident) 909 keV gamma ray photon in 100% of disintegrations. These characteristics, coupled with protracted antibody bio-kinetics, lead to relatively high radiation doses to patients. For example, the ^89^Zr-labeled anti-PSMA monoclonal antibody J591 ([^89^Zr]Zr-DFO-J59) delivers a 25 times higher whole-body radiation dose (mGy/MBq) than 2-[^18^F]FDG [9, 10]. There is thus an incentive to reduce administered activities. Our experience at MSKCC reflects this; initial ^89^Zr-immunoPET studies featured administered activities of approximately 185 MBq (5 mCi) [9, 11–13], while more recent work has used approximately 74 MBq (2 mCi) [4, 5, 14]. In other centers, even lower activities of approximately 37 MBq (1 mCi) [1, 6] have been used.

Reducing administered activity may lead to a reduction in image quality. In addition, ^89^Zr-immunoPET agents generally have lower image quality in comparison to ^18^F-tracers due to constraints on administered activity, lower positron yield, longer post-administration imaging times and practical limits on acquisition times. For these reasons, the use of modern image reconstruction methods is especially important for image optimization [15].

PET image quality may be improved using reconstruction algorithms that decrease noise and/or increase spatial resolution. One such method, Q.Clear (GE Healthcare), a Bayesian penalized likelihood reconstruction algorithm, is designed to minimize image noise while allowing full convergence, thus increasing the signal-to-noise ratio [16–18]. Noise is suppressed by the use of a penalty term, a function of the difference between neighboring voxels and their sum [19]. A factor (termed β) controls the relative strength of the penalty function and is the only selectable input variable to the algorithm [16, 19–22]. Q.Clear uses a block sequential regularized expectation maximization (BSREM) algorithm that allows each voxel to achieve full convergence, potentially providing a more accurate SUV [16]. This contrasts with conventional ordered-subset expectation maximization (OSEM) that has to be stopped after a few iterations in order to prevent excessive image noise [23]. Previous studies have shown that Q.Clear improves lesion signal-to-noise ratio (SNR) for both 2-[^18^F]FDG PET/CT [16–18, 21, 24] and [^68^Ga]Ga-RM2/[^68^Ga]Ga-PSMA-11 PET/MRI [25] compared to OSEM reconstruction.

For immunoPET imaging, the key clinical issue is to minimize lesion misidentification (either false positive or false negative) resulting from image noise or excessive smoothing. Image quality should be interpreted in this context, and although quantitative accuracy is desirable, it is less critical. Radiation dosimetry is an important characteristic of an immunoPET agent and largely sets how much can be administered to patients, but it is not necessary to estimate this on a patient-by-patient basis, as it would be for a therapeutic agent. Indeed, for most patients, immunoPET imaging consists of a single scan at typically 5–7 days post administration, and dosimetry, based on the areas under activity-time curves, is not an option. Other aspects of immunoPET quantification such as lesion SUV may, in the future, have utility in longitudinal studies of disease response but, at present, these types of study are largely precluded by the relatively high radiation doses produced by immunoPET agents. In order to facilitate such longitudinal studies, radiation doses would have to be reduced by minimizing administered activities and employing advanced reconstruction techniques. In this paper, our goal was to evaluate the use of Q.Clear for ^89^Zr-immunoPET image reconstruction and its potential to improve image quality and to facilitate a reduction in administered activity of ^89^Zr-immunoPET tracers.

## Material and methods

### Patients

This was a retrospective study of ^89^Zr-immunoPET/CT scans that were accrued on three prospective clinical trials (NCT02286843, NCT03665155, and NCT02675829) [2–5, 26]. All clinical studies were performed in compliance with the Health Insurance Portability and Accountability Act and with Institutional Review Board approval and patient informed consent. A total of eight ^89^Zr-immuno-PET scans (4 men, 4 women, mean age 65.5 years, range 41.5–80.5 years) were selected based on the presence of PET positive lesions and the availability of stored list mode data (Table 1). These eight scans included four [^89^Zr]Zr-DFO-daratumumab scans for CD38-targeted imaging of myeloma, two [^89^Zr]Zr-DFO-pertuzumab scans for HER2-targeted imaging of breast cancer, and two [^89^Zr]Zr-DFO-trastuzumab scans for HER2 targeted imaging of lung cancer.

**Table 1:** Details of eight ^89^Zr-immunoPET patients

Scan #	Tracer	Sex	Weight in kg	Administered activity in MBq	Malignancy	Analyzed tracer avid lesions
1	^89^ZR-Daratumumab	M	87.5	66.2	Multiple Myeloma	2nd Rib right, Os ileum right, Vertebrae L3
2	^89^ZR-Daratumumab	F	85.3	68.0	Multiple Myeloma	Mandibula right, Clavicle right, Os ilium right
3	^89^ZR-Daratumumab	M	90.2	65.0	Multiple Myeloma	Femora left, Scapula left, 7th Rib left, Femora left
4	^89^ZR-Daratumumab	M	110.7	68.3	Multiple Myeloma	Os ileum right, Os sacrum, Soft tissue left shoulder
5	^89^ZR-Pertuzumab	F	66.0	70.7	Breast Cancer	Vertebrae Th12, Femora left, Soft tissue retroperitoneal
6	^89^ZR-Pertuzumab	F	66.0	71.4	Breast Cancer	Os ileum right, Os ischii right
7	^89^ZR-Trastuzumab	F	52.0	67.0	Lung Cancer	Os ilium left, Spleen
8	^89^ZR-Trastuzumab	M	75.0	75.9	Lung Cancer	Vertebrae T11, Node portocaval, Vertebrae T11, Scapula right

### PET/CT and reconstruction

Patients were imaged at a mean time of 6.3 days (range 5.0–8.0) following administration of a mean of 69 MBq (range 65–76) ^89^Zr-immunoPET tracer. Whole body (WB) images were acquired from skull apex to midthigh on a dedicated research PET/CT scanner (GE Discovery 710; lutetium yttrium orthosilicate (LYSO) crystals, measured system sensitivity 7.1 cps/kBq) in 3-dimensional mode with a mean emission time per bed position of 6.9 min (range 6.0–8.0). At the time of imaging, the mean total activity in the WB PET images was 14 MBq (range 9–17). Low-dose CT scans were acquired with an x-ray tube current of 80 mA. Images were first reconstructed using our standard clinical parameters featuring ordered subset expectation maximization (OSEM) into a 128 × 128 matrix; voxel size 0.098 ml (5.47 × 5.47 × 3.27 mm) with 2-iterations, 16-subsets, a 6.4-mm Gaussian transaxial filter, “heavy” *z*-axis filtering, and all manufacturers’ corrections active (CT-based attenuation, scatter, time-of-flight (TOF), and point-spread-function (PSF)). Subsequently, list-mode PET data for each patient was time-binned to include 100% (no counts discarded), 50% (50% of counts discarded), and 25% (75% of counts discarded) of the original counts. The standard OSEM reconstruction with 100% of original counts was used as a reference for comparison to all other reconstructions. Additionally, 100% count data were reconstructed using the Q.Clear BSREM algorithm into a 256 × 256 matrix; voxel size 0.024 ml (2.73 × 2.73 × 3.27 mm) with incremental *β-*values of 150, 300, 600, 1000, 1600, 2400, 3600, 5200, and 7200. For the simulated reduced doses (corresponding to 25% and 50% of the original counts), incremental *β-*values of 1600, 2400, 3600, 5200, and 7200 were used.

The rationale for choosing the abovementioned reconstructions was twofold: firstly, to evaluate the optimal *β-*value for the current ^89^Zr-immuno-PET clinical workflow, and secondly, to evaluate the possibility of decreasing administered activity while maintaining image quality due to improved reconstruction.

### Image analysis

#### Clinical assessment

Image reading was performed by a radiologist dually boarded in diagnostic radiology and nuclear medicine with 15 years of experience in PET/CT including multiple ^89^Zr-immunoPET tracers (GAU) and a radiologist with 5 years of experience in PET/CT imaging (JK) in consensus. Readers were not informed of the nature of the data. Different reconstructed images of each patient were presented separately but in random order to the readers in a single reading session, so the readers were aware of their previous evaluation. However, retrospective changes in evaluation were not allowed. All PET/CT data sets for each patient were interpreted with respect to lesion detectability and subjective image quality, applying a 5-point ordinal (see Table 2). Thereafter, average values were calculated for each ^89^Zr-immuno-PET agent.

**Table 2:** Definition of the applied 5-point ordinal scale for image quality and lesion detectability

Rating	Criteria
1	Non-diagnostic: inability to discern lesions from background
2	Poor: only subtle distinction of lesions from background
3	Moderate: ability to discern lesions with significant noise
4	Good: ability to discern lesions with low noise
5	Excellent: ability to discern lesions without noise

### Objective measurements

Volumes of interest (VOI) were drawn round index lesions and maximum standardized uptake values (SUV_max_) measured. In addition, VOI were drawn within the liver, excluding gross anatomical or uptake heterogeneities, and the mean (SUV_mean_) and standard deviation (σ_SUV_) used to derive a liver signal-to-noise ratio (SNR) according to *SNR* = *SUV*_*mean*_/*σ*_*SUV*_. For each patient, VOIs were drawn on an image set that was subjectively judged to be most clear using Hybrid Viewer Gold Client v2.3 (HERMES Medical Solutions AB, Stockholm, Sweden) and copied to all other image sets.

## Results

### Image features for different *β*

**Fig. 1:**
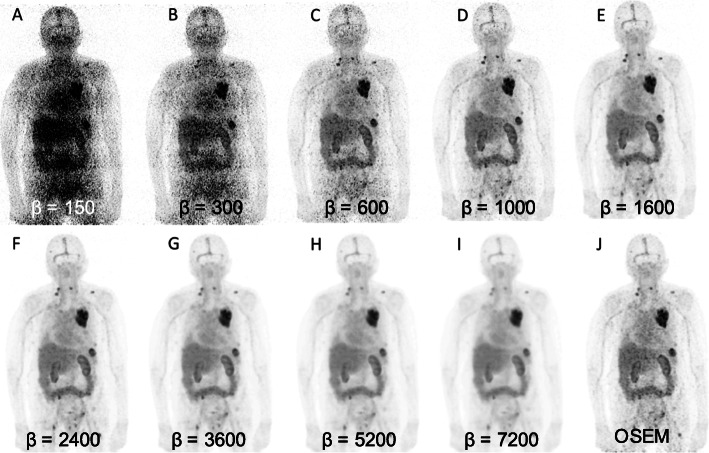
MIP images of [^89^Zr]Zr-DFO-trastuzumab reconstructed using 100% of original counts with Q.Clear BSREM and *β*-values of 150, 300, 600, 1000, 1600, 2400, 3600, 5200, and 7200 (**a**–**i**, respectively) together with the standard OSEM reconstruction (**j**)

Figure 1 is an example of a patient imaged with [^89^Zr]Zr-DFO-trastuzumab that illustrates the effects of using different values of *β* in Q.Clear BSREM reconstruction. Images reconstructed with *β-*values in the range 150–600 (Fig. 1, a–c) were noisier and of inferior quality to the OSEM reference (Fig. 1, j) while *β-*values of 1000 generated images (Fig. 1, d) approximately similar to OSEM. Q.Clear image quality improved with increasing *β* from 1600 to 3600 (Fig. 1, e–g). For *β-*values of 5200–7200 (Fig. 1, h, i), images became over-smoothed and lesions lost conspicuity.

### Subjective evaluation of image quality

**Fig. 2:**
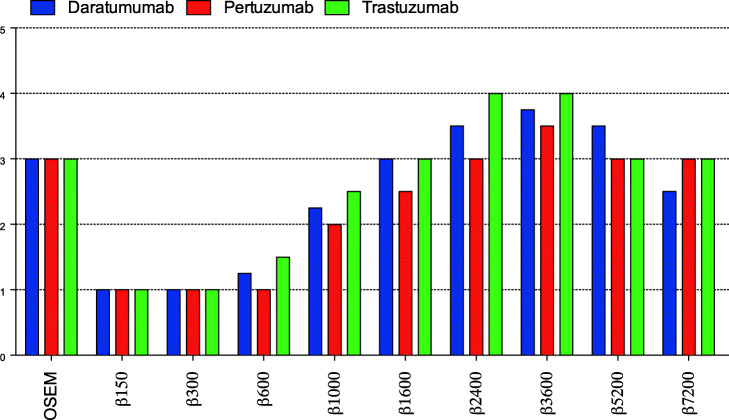
Subjective evaluation of image quality for three ^89^Zr-immunoPET agents using standard OSEM reconstructions and Q.Clear BSREM with increasing *β*-values. See Table 2 for the description of the 5-point scale. Q.clear reconstructions with a *β*-value of 3600 provided superior image quality to standard OSEM reconstructions for all three ^89^Zr-immunoPET tracers

Standard OSEM reconstructions were rated as “moderate” (mean score: 3 out of 5) for all three ^89^Zr-immunoPET agents. In general, Q.Clear images with low *β-*values (150–600) were deemed inferior in quality to OSEM, with image quality improving around *β* = 1000 and being approximately equivalent to OSEM for *β* = 1600. Image quality continued to improve up to *β* = 3600, where the Q.Clear reconstructions of all three ^89^Zr-immunoPET agents were judged to be superior to standard OSEM reconstruction. At *β* = 3600, the mean image scores were 4 out of 5 for [^89^Zr]Zr-DFO-trastuzumab, 3.75 out of 5 for [^89^Zr]Zr-DFO-daratumumab, and 3.5 out of 5 for [^89^Zr]Zr-DFO-pertuzumab. For higher *β-*values (5200, 7200), image quality evaluation decreased to approximately the level of the standard OSEM reconstructions (Fig. 2).

**Fig. 3:**
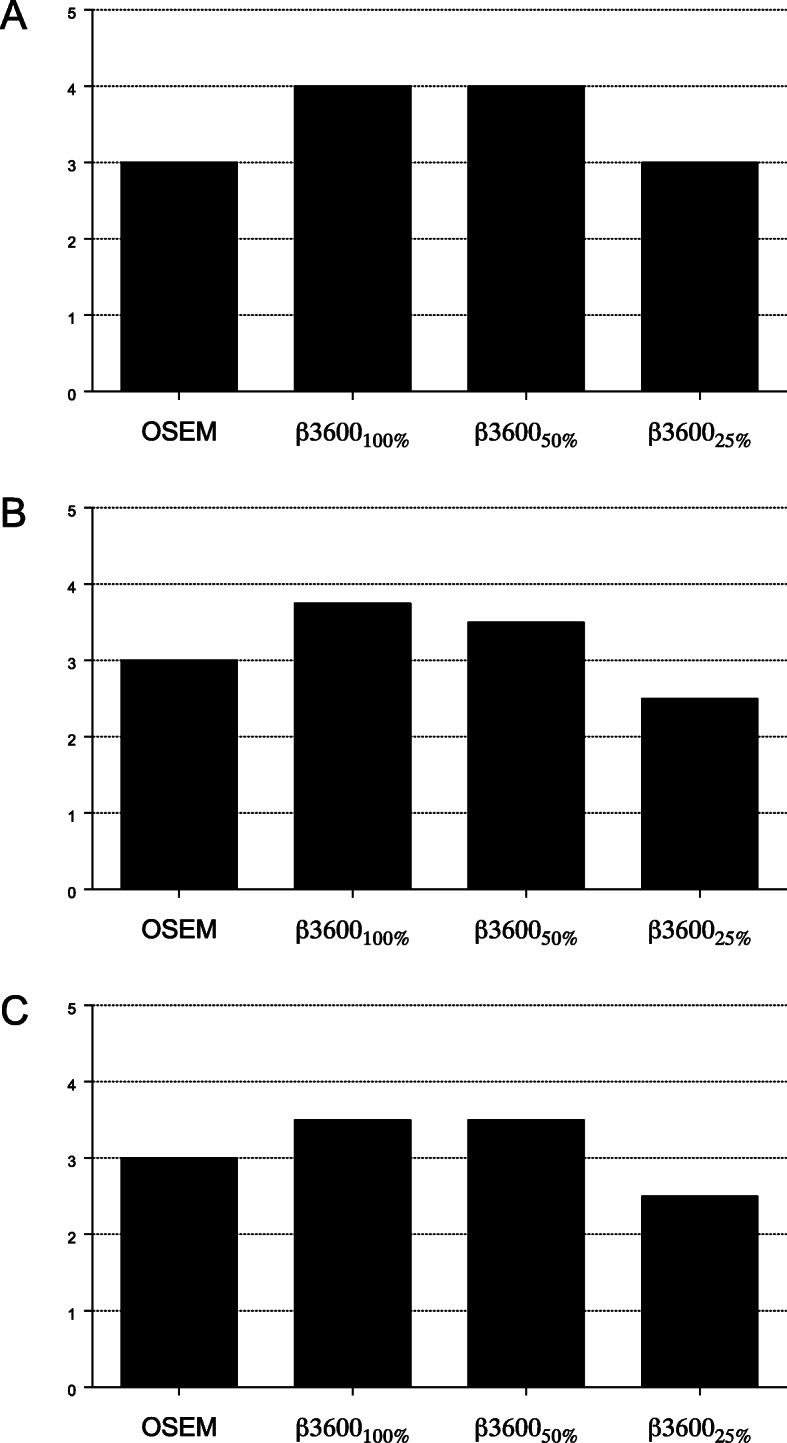
Scores for image quality and lesion detectability for **a** [^*89*^Zr]Zr-DFO-trastuzumab, **b** [^*89*^Zr]Zr-DFO-daratumumab, and **c** [^*89*^Zr]Zr-DFO-pertuzumab using OSEM reconstructions and Q.Clear BSREM with a *β*-value of 3600 for different fractions of original counts, applying a 5-point ordinal scale

**Fig. 4:**
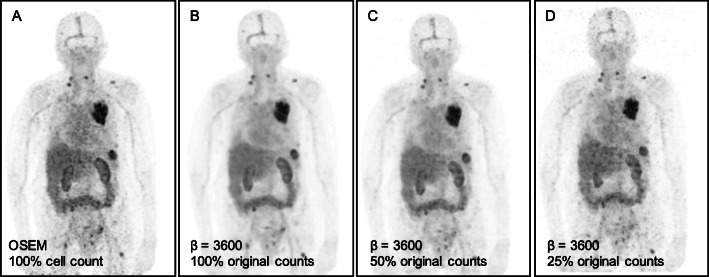
MIP images of a [^*89*^Zr]Zr-DFO-trastuzumab patient using **a** OSEM reconstruction with 100% original counts and using Q.Clear BSREM with *β* = 3600 for **b** 100% original counts, **c** 50% original counts, and **d** 25% original counts, respectively. For 100% of counts, Q.Clear produced images with improved signal-to-noise compared to standard OSEM. Even at only 25% of counts, Q.Clear produced images of comparable quality to OSEM at 100% of counts

To simulate reduced administered tracer activity, events were discarded in each list mode stream to reduce counts to 50% and 25% of the original values and Q.Clear BSREM reconstructions were performed using *β-*values of 1600 and higher. As expected, as counts decreased from 100 to 50%, and again from 50 to 25%, there was a reduction in image quality. Of note, for Q.Clear with *β-*values between 2400 and 5200, image quality using only 50% of counts was greater than or equal to standard OSEM performed with 100% of counts (Fig. 3). For example, for [^89^Zr]Zr-DFO-trastuzumab, Q.Clear with *β* = 3600 and only 50% of original true counts produced images with a mean score of 4, compared with a mean score of 3 for standard OSEM reconstructions and 100% of counts. Similar improvements with Q.Clear at these specifications were seen in [^89^Zr]Zr-DFO-daratumumab and [^89^Zr]Zr-DFO-pertuzumab images (mean score of 3.5, respectively). Even with only 25% original counts, Q.Clear BSREM images of [^89^Zr]Zr-DFO-trastuzumab and [^89^Zr]Zr-DFO-pertuzumab with *β-*values of 3600–5200 and images of [^89^Zr]Zr-DFO-daratumumab with *β* = 5200 produced image quality similar to standard OSEM images with 100% counts. Figure 4 demonstrates an example of [^89^Zr]Zr-DFO-trastuzumab images reconstructed with standard OSEM (100% counts) and Q.Clear BSREM with *β* = 3600 for 100%, 50%, and 25% of original counts.

### Objective metrics of image quality

#### SUVmax

**Fig. 5:**
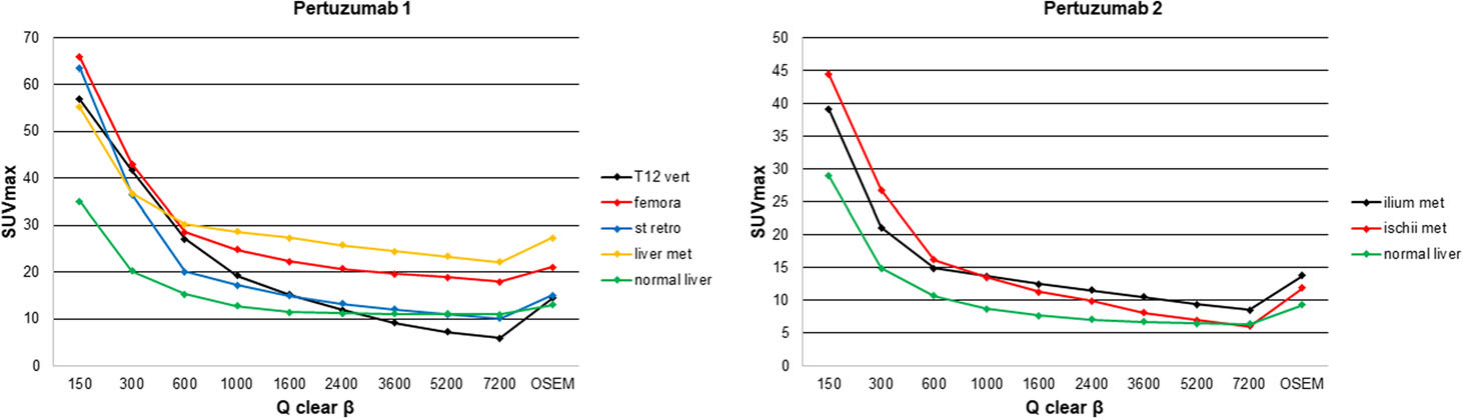
SUVmax values for metastases and normal liver for [^*89*^Zr]Zr-DFO-pertuzumab images as *β*-value increases from 150 to 7200. OSEM SUVmax also shown

All estimates of SUVmax (lesions and normal liver) were maximal for *β* = 150 and decreased monotonically with increasing *β*. Figure 5 demonstrates how lesion and liver SUVmax for [^89^Zr]Zr-DFO-pertuzumab varied with *β*. The very high values of SUVmax for low values of *β* are a reflection of excessive image noise. For the [^89^Zr]Zr-DFO-pertuzumab cases of Fig. 5, Q.Clear SUVmax are approximately equivalent to OSEM for *β-*values in the range 1000–1600. SUVmax continued to decrease as *β* increased and images become progressively smoother. Similar patterns of changing SUVmax were also observed for [^89^Zr]Zr-DFO-daratumumab and [^89^Zr]Zr-DFO-trastuzumab (see Table 3 and supplemental Figures S2, S3).

**Table 3:**
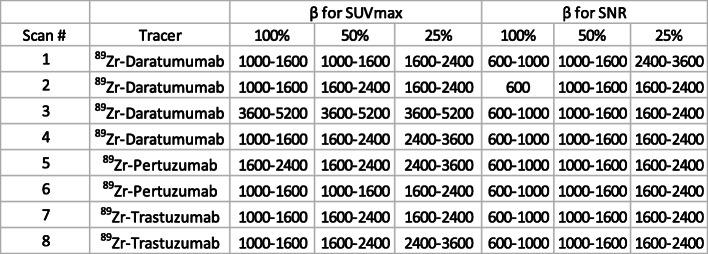
Q.Clear *β* parameters that produce SUVmax and liver SNR equal to that of OSEM reconstruction using 100%, 50% and 25% of counts

#### Liver SNR

The signal-to-noise ratio for liver VOI increased monotonically with increasing *β* for Q.Clear reconstruction. Figure S1 illustrates how liver SNR varied for [^89^Zr]Zr-DFO-pertuzumab with *β* for 100% counts. Q.clear SNR was greater than the corresponding OSEM values for *β ≥* 1000. For reduced count reconstructions, Q.clear SNR were greater than full-count OSEM for *β ≥* 1600 (50% counts) and *β ≥* 2400 (25% counts). Again, similar patterns of changing SNR were observed for [^89^Zr]Zr-DFO-daratumumab and [^89^Zr]Zr-DFO-trastuzumab (see Table 3 and supplemental Figures S2, S3).

## Discussion

This retrospective analysis of ^89^Zr-immuno-PET clinical image data demonstrated that Q.Clear BSREM PET/CT image reconstruction using a *β-*value of 2400–3600 increased image quality compared to standard OSEM reconstruction. In addition, the Q.Clear algorithm enabled lower count datasets to generate images of comparable quality to full-count OSEM images. We examined discarding 50% and 75% of the original counts as a means of mimicking 50% and 25% of the administered activity and found comparable image quality to full-count OSEM following Q.Clear reconstruction with *β-*values in the range 3600–7200. This provides a means to increase the image quality of future ^89^Zr-immuno-PET studies and/or decrease the administered activity of ^89^Zr-labeled tracer.

Despite the improvements achieved by Q.Clear BSREM, no images in this study were assessed as 5 on the ordinal scale of image quality, representing the ability to discern lesions without apparent noise. Even the best rated pictures with 100% counting and *β* = 3600 had some noise while providing adequate lesion contrast and were therefore rated with 4 out of 5 points. Due to the associated physical and biokinetic constraints, ^89^Zr-immuno-PET images are unlikely to provide equivalent image quality to ^18^F or ^68^Ga tracers [27, 28].

In addition to subjective image quality, we examined the objective measurements of lesion and liver SUVmax and liver SNR for all reconstructions. Low values of *β* (150–600) were associated with very high SUVmax which tended to stabilize around *β = 1000–1*600 for 100% counts and at higher values for the reduced count images. Such objective measures are important for the assessment of PET/CT studies [29] and indicate that SUVmax values for metastases are likely to be overestimated for lower *β-*values. Liver SNR generally increased with *β* all the way up to the 5200–7200 range, reflecting a progressive increase in image smoothness. The potential downside to excessive image smoothing is a consequential reduction in lesion SUVmax and/or contrast recovery and compromised lesion detectability. However, notwithstanding the utility of objective measurements, we found that the clinical read and interpretation was the most reliable guide to image quality.

It is noteworthy that the *β-*values used in this study are much larger than those found suitable for improving image quality for ^18^F-labeled tracers; these are typically in the 150–400 range [30]. This is a consequence of the very low effective activities present at the time of imaging. For our patients, the average WB activity was 14 MBq and, considering the 23% positron yield of ^89^Zr, would be equivalent to only 3.2 MBq (0.09 mCi) of ^18^F. Obviously, the reduced count datasets would be equivalent to 50% and 25% of this value. Clinical images were acquired with an average emission time per bed position of 7 min. For a typical 8–9 bed WB PET scan, this is approximately 1 h of emission data collection and is at the upper limit of what is possible from the perspectives of both clinic logistics and patient compliance.

The relatively high radiation absorbed doses produced by ^89^Zr-immunoPET could impede more widespread clinical implementation. In particular, reducing absorbed dose is a prerequisite for particularly desirable sequential imaging studies (e.g., response assessment, post-therapy surveillance) and for pediatric imaging applications. The only way to reduce the radiation dose is to reduce the administered activity. This study suggests that with Q.Clear BSREM and an appropriate choice of *β-*value, it may be possible to reduce the administered activity of ^89^Zr-immunoPET tracer to 18.5 MBq (0.5 mCi).

Limitations to this work include the fact that it was a single-center study with a small number of patients and its further subdivision into three categories led to a reduced statistical power. Patients were selected based on the presence of metastatic disease and the availability of stored data for the additional reconstructions.

## Conclusion

Q.Clear BSREM reconstructions improved the quality of ^89^Zr-immunoPET images compared with standard OSEM reconstructions. For administered activities of approximately 74 MBq and images acquired approximately 1 week post-administration, a *β-*value of 3600 appears a reasonable choice for optimal image reconstruction. These findings can be applied to future ^89^Zr-immunoPET studies to improve image quality and may enable a reduction in the administered activity.

## Supplementary Information

**Additional file 1:Figure S1**. Liver SNR values for [^89^Zr]Zr-DFO-pertuzumab Q.Clear images using 100%, 50% and 25% of original counts as β-value increases from 150 to 7200. OSEM (100% counts) SNR also shown. Q.Clear SNR was greater than the corresponding OSEM values for β ≥ 1000. For reduced count reconstructions, Q.Clear SNR were greater than full-count OSEM for β ≥ 1600 (50% counts) and β ≥ 2400 (25% counts).

**Additional file 2:Figure S2.** SNR and SUVmax values for two sets of [^89^Zr]Zr-DFO-trastuzumab images as β-value increases from 150 to 7200.

**Additional file 3:Figure S3**. SNR and SUVmax values for four sets of [^89^Zr]Zr-DFO-daratumumab images as β-value increases from 150 to 7200.
