# Supplementary figures and images for: Improved image reconstruction of 89Zr-immunoPET studies using a Bayesian penalized likelihood reconstruction algorithm
Source: EJNMMI Phys. 2021 Jan 19;8:6. doi: 10.1186/s40658-021-00352-z (PMC7815860; doi:10.1186/s40658-021-00352-z)

Pertuzumab 1

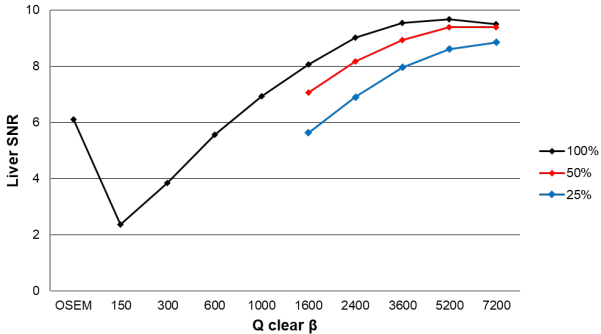

Pertuzumab 2

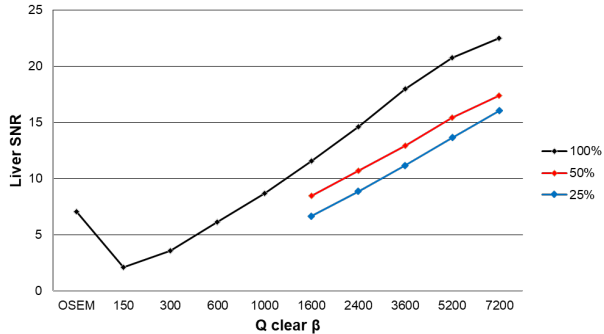

Supplement: Supplementary file 1 — Additional file 1:Figure S1. Liver SNR values for [89Zr]Zr-DFO-pertuzumab Q.Clear images using 100%, 50% and 25% of original counts as β-value increases from 150 to 7200. OSEM (100% counts) SNR also shown. Q.Clear SNR was greater than the corresponding OSEM values for β ≥ 1000. For reduced count reconstructions, Q.Clear SNR were greater than full-count OSEM for β ≥ 1600 (50% counts) and β ≥ 2400 (25% counts). [file 40658_2021_352_MOESM1_ESM.pdf]

Trastuzumab 1

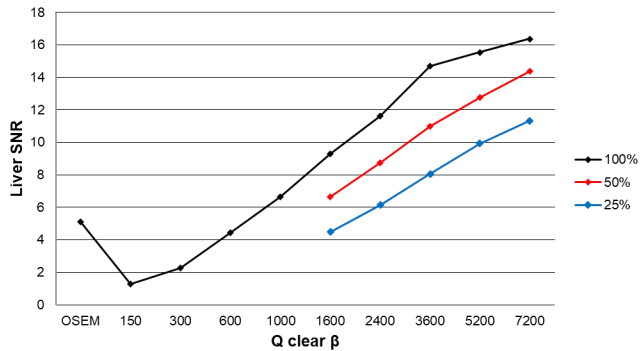

Trastuzumab 1

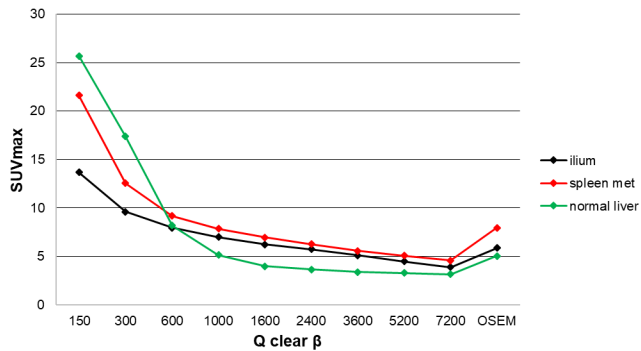

Trastuzumab 2

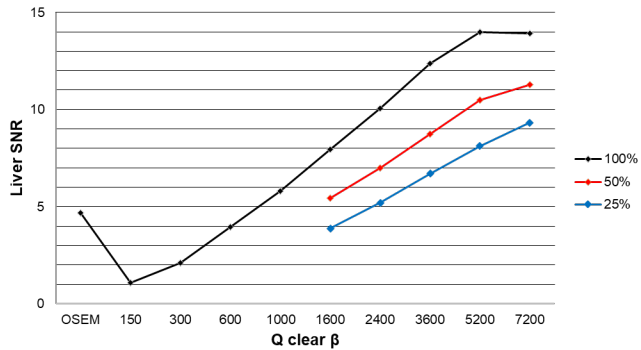

Trastuzumab 2

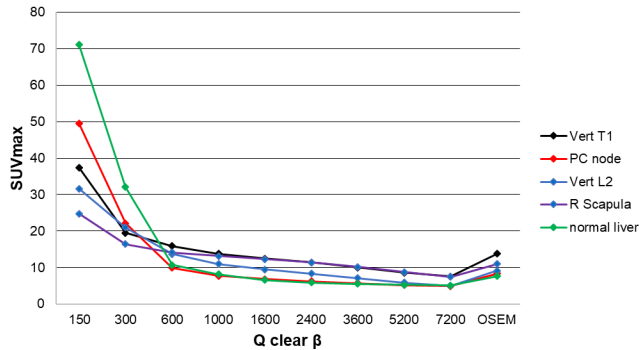

Supplement: Supplementary file 2 — Additional file 2:Figure S2. SNR and SUVmax values for two sets of [89Zr]Zr-DFO-trastuzumab images as β-value increases from 150 to 7200. [file 40658_2021_352_MOESM2_ESM.pdf]
