# Supplementary material for: Improved image reconstruction of 89Zr-immunoPET studies using a Bayesian penalized likelihood reconstruction algorithm
Source: EJNMMI Phys. 2021 Jan 19;8:6. doi: 10.1186/s40658-021-00352-z (PMC7815860; doi:10.1186/s40658-021-00352-z)
Supplement: Supplementary file 3 — Additional file 3:Figure S3. SNR and SUVmax values for four sets of [89Zr]Zr-DFO-daratumumab images as β-value increases from 150 to 7200. [file 40658_2021_352_MOESM3_ESM.pdf]

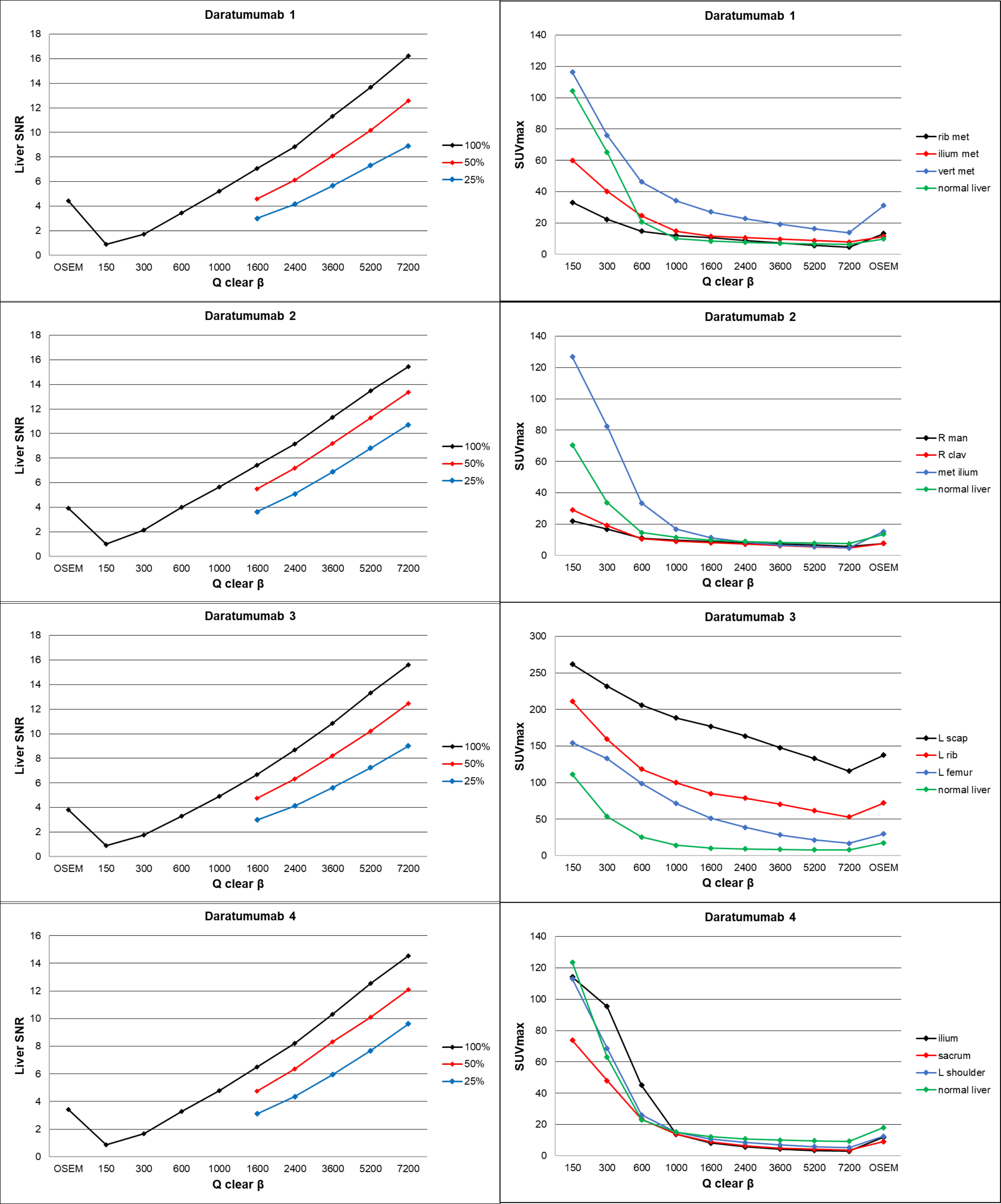
Daratumumab 1
Liver SNR
Q clear β
OSEM
150
300
600
1000
1600
2400
3600
5200
7200
100%
50%
25%
Daratumumab 1
SUVmax
Q clear β
rib met
ilium met
vert met
normal liver
Daratumumab 2
Liver SNR
Q clear β
100%
50%
25%
Daratumumab 2
SUVmax
Q clear β
R man
R clav
met ilium
normal liver
Daratumumab 3
Liver SNR
Q clear β
100%
50%
25%
Daratumumab 3
SUVmax
Q clear β
L scap
L rib
L femur
normal liver
Daratumumab 4
Liver SNR
Q clear β
100%
50%
25%
Daratumumab 4
SUVmax
Q clear β
ilium
sacrum
L shoulder
normal liver
